# Supplementary material for: Heart-Focused Anxiety, General Anxiety, Depression and Health-Related Quality of Life in Patients with Atrial Fibrillation Undergoing Pulmonary Vein Isolation
Source: J Clin Med. 2022 Mar 22;11(7):1751. doi: 10.3390/jcm11071751 (PMC8999774; doi:10.3390/jcm11071751)
Supplement: Supplementary file 1 [file jcm-11-01751-s001.zip › jcm-1613513-supplementary.pdf]

Supplementary Materials

Table S1. Cut-off scores for the Cardiac Anxiety Questionnaire (CAQ).

		Age dependent			
		18-43	44-54	55-66	67-92
**CAQ**	**0-4**				
Total (male)		0.94	1.41	1.70	1.92
Total (female)		1.21	1.35	1.82	2.00
Fear (male)		1.25	1.50	1.88	1.96
Fear (female)		1.50	1.50	2.00	2.13
Attention (male)		1.00	1.60	2.00	2.10
Attention (female)		1.20	1.40	1.82	2.20
Avoidance (male)		1.25	1.50	2.00	2.75
Avoidance (female)		1.50	2.00	2.00	2.75

CAQ: Cardiac Anxiety Questionnaire. Cut-off scores refer to Fischer et al. for CAQ [15].
